# Barriers to prescribing oral anticoagulants to inpatients aged 80 years and older with nonvalvular atrial fibrillation: a cross-sectional study

**DOI:** 10.1186/s12877-022-02965-0

**Published:** 2022-03-30

**Authors:** Xin Xia, Lishuang Wang, Taiping Lin, Jirong Yue, Zhonghua Yang, Chongqing Mi, Zaibo Liao, Yanyu Chen, Ning Ge, Chenkai Wu

**Affiliations:** 1 The Center of Gerontology and Geriatrics and National Clinical Research Center for Geriatrics, West China Hospital, Sichuan University, Chengdu, Sichuan Province China; 2 College of Pharmacy, Macau University of Science and Technology, Macau, China; 3 Shenqiu County People’s Hospital, Zhoukou, China; 4 Global Health Research Center, Duke Kunshan University, Kunshan, Jiangsu China

**Keywords:** Atrial fibrillation, Oral anticoagulants, Antithrombotic, Geriatrics

## Abstract

**Background:**

To investigate the temporal trend of the prevalence of underprescription of anticoagulation treatment and explore the factors associated with underprescription of oral anticoagulants (OACs) among inpatients aged ≥ 80 years with nonvalvular atrial fibrillation (NVAF).

**Methods:**

We retrospectively reviewed the medical records of inpatients with a discharge diagnosis of NVAF from a medical database. We used the Pearson chi-square or Fisher’s exact test to compare categorical variables between patients with and without OAC prescriptions during hospitalization. Logistic regression analysis was used to assess the association between risk factors and underprescription of OACs.

**Results:**

A total of 4375 patients aged ≥ 80 years with AF were assessed in the largest academic hospital in China from August 1, 2016, to July 31, 2020, and 3165 NVAF patients were included. The prevalence of underprescription of OACs was 79.1% in 2017, 71.3% in 2018, 64.4% in 2019, and 56.1% in 2020. Of all participants, 2138 (67.6%) were not prescribed OACs; 66.3% and 68.2% of patients with and without prior stroke did not receive OACs, respectively. Age (85–89 vs 80–84, OR = 1.48, 95% CI (1.25–1.74); 90 + vs 80–84, OR = 2.66, 95% CI: 2.09–3.42), clinical department where patients were discharged (Reference = Cardiology, Geriatrics: OR = 2.97, 95% CI: 2.45- 3.61; neurology: OR = 1.25, 95% CI: 0.96, 1.63; others: OR = 4.23, 95% CI: 3.43- 5.24), use of antiplatelets (OR = 1.69, 95% CI: 1.45- 1.97), and history of stroke (OR = 0.83, 95% CI: 0.71- 0.98 adjusted age), and dementia (OR = 2.16, 95% CI: 1.60- 2.96) were significantly associated with not prescribing OACs.

**Conclusions:**

The prevalence of underprescription of OACs has decreased over the past several years. The rate of underprescription of OACs was higher among NVAF patients who were older, prescribed antiplatelets, discharged from nondepartmental cardiology, and suffered from comorbidities. This study found iatrogenic factors affecting the underprescription of OACs in inpatients aged ≥ 80 years, providing clues and a basis for the standardized use of OACs in inpatients.

## Background

Nonvalvular atrial fibrillation (NVAF), a common type of atrial fibrillation (AF) that can cause stroke and other serious complications, is prevalent among older adults in China. The number of older adults with AF in China is projected to be twice as high as that in the US by 2050 [1, 2]. Stroke is one of the most serious complications associated with AF and affects approximately one-third of older AF patients in China [3].

Antithrombotic agents have been consistently shown to be effective in reducing the risk of stroke among patients with AF. Although there has been a surge in the number of prescriptions for anticoagulants since direct and nonvitamin K antagonist oral anticoagulants (DOACs/NOACs) entered the Chinese market in 2009 and 2013, respectively, anticoagulation medications were still severely underutilized among patients with NVAF, with a prescription rate of less than 30% [4–7]. It is therefore important to identify risk factors for low utilization of anticoagulation treatment in older patients who have NVAF.

The purpose of the present study were to investigate the temporal trend of the prevalence of underprescription of anticoagulation treatment and to identify the factors that may contribute to underprescription of anticoagulation among NVAF patients aged 80 years and older.

## Methods

### Participants

This retrospective study was carried out in a tertiary teaching hospital with over 4,000 beds in western China. The data were extracted from the hospital database and anonymized between August 1, 2016, and July 31, 2020. The study was approved by the Ethical Review Committee of West China Hospital of Sichuan University with the committee’s reference number 2015(150). The ethical committee approved the research protocol and waived the need for informed consent because of the retrospective nature of this study, and waiving informed consent will not adversely affect the rights and health of the study subjects. The regulations followed the Declaration of Helsinki and ethical review of biomedical research involving people in China.

The study included participants who aged 80 years and over with any diagnosis of AF during hospitalization and excluded participants who (i) underwent invasive procedures; (ii) absolute contraindications to anticoagulation included moderate to severe hypertension (blood pressure ≥ 160/100 mmHg), with a diagnosis of liver function impairment, coagulopathy accompanied by bleeding tendency, active peptic ulcer and hemorrhagic diseases; with a diagnosis of intracranial tumor; and (iii) mitral valve stenosis or previous prosthetic valve replacement.

### Data collection

Patient information was extracted from their medical records during hospitalization by trained hospital personnel. Demographics included age and sex. Clinical variables included departments where they were discharged from, years of hospitalization, history of diabetes, medical conditions (hypertension and blood pressure data, congestive heart failure, prior stroke, coronary heart disease (CHD), thromboembolic events, atherosclerosis, liver and renal function, medications administered during hospitalization, and bleeding events during hospitalization), the international normalized ratio (INR), and the frequency of monitoring, antithrombotic medications administered, and all the comorbidities to calculate the Charlson comorbidity index (CCI) [8, 9], including dementia, diabetes, myocardial infarction, etc. All methods were carried out in accordance with relevant guidelines and regulations.

### Thromboembolic and bleeding risk assessment

We estimated the risk of stroke using the CHA_2_DS_2-_VASc, a common method for predicting thromboembolic risk in patients with AF [10–15]. Seven risk factors were included: heart failure, hypertension, age, diabetes, history of stroke/transient ischemic attack (TIA), vascular diseases (peripheral arterial disease, previous myocardial infarction, and aortic atheroma), and female sex. Each risk factor receives one point except age $\ge$ 75 years and history stroke/TIA, which receive 2 points. The total CHA_2_DS_2-_VASc score ranges from 0 to 9 (highest risk).

We evaluated the risk of bleeding using the [16, 17], in which 1 point was assigned to uncontrolled hypertension (systolic blood pressure > 160 mmHg), abnormal renal or liver function (abnormal liver function was defined as bilirubin concentration in plasma was higher than 2 times the upper limit of the normal range, or one of glutamic-pyruvic transaminase, glutamic-oxalacetic transaminase, and alkaline phosphatase level in plasma was higher than 3 times the upper limit of normal range; renal dysfunction was defined as serum creatinine level was ≥ 200 mmol/l, or with the diagnosis abnormal liver or kidney function), previous stroke, bleeding history, labile international normalized ratio defined as time in therapeutic range (TTR) < 60%, age > 65 years, and use of drugs (antiplatelet/anticoagulation) or alcohol (> 8 drinks/week). A HAS-BLED score of 3 points or more indicates a high risk of bleeding.

### Antithrombotic medications review

We identified antithrombotic medications administered in elderly patients with NVAF from the electronic medical chart included all information of prescriptions during hospitalization, as well as discharge orders, even for patients discharged within 24 h. during the hospital stay, which consisted of three types: oral anticoagulants (OACs), lower molecular weight heparins (LMWH) or heparin, and antiplatelets. OACs consisted of vitamin K antagonists (VKAs) and DOACs/NOACs. NOACs include dabigatran, rivaroxaban, and apixaban, which have been gradually introduced to China since 2012. Antiplatelet therapy involved the use of aspirin, clopidogrel, ticlopidine, ticagrelor, dipyridamole, and tirofiban. Someone prescribed an antiplatelet, LMWH or heparin and then switched to an OAC was assigned to OACs.

### Statistical analysis

We used the mean and SDs or medians and interquartile ranges to describe continuous variables and counts and percentages to describe count variables of the entire sample. Baseline characteristics of patients were compared and summarized by means of the independent-sample t test for continuous variables and the Pearson chi-square test or Fisher’s exact test for categorical variables between patients with and without prescription of OACs. Subsequently, we used both univariate and multivariable logistic regression analyses adjusting for confounders to identify the factors that were associated with underprescription of anticoagulation. To test for differences in stroke on anticoagulants, we stratified for stroke and then performed logistic regression analysis using the same method as above.

The cut-off of the *P* value for testing significance was set at 0.05. All statistical analyses were conducted using the R Project for Statistical Computing (R-4.0.5-win, University of Science and Technology of China; 2021–03-31).

## Results

### Sample characteristics

**Fig. 1:**
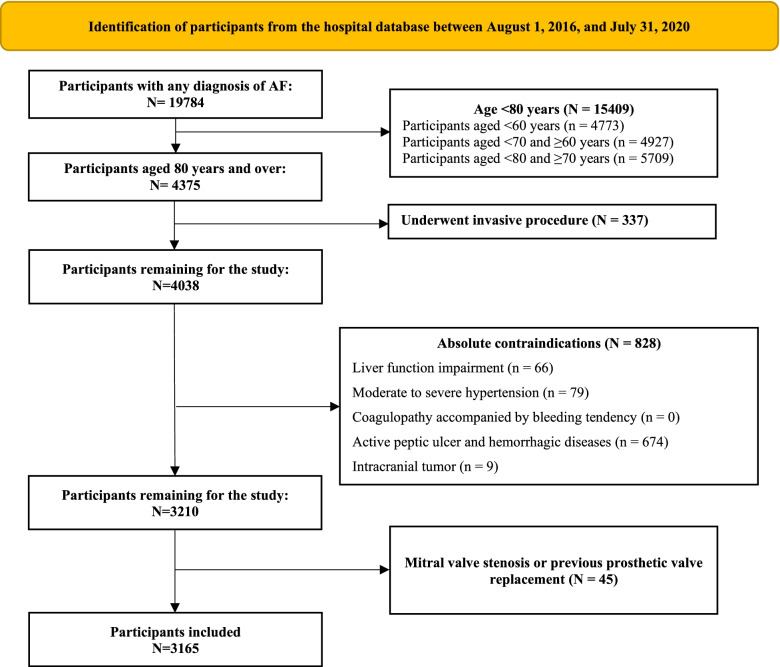
Flow diagram of the selection of the analytic sample

A total of 4375 participants with any diagnosis of AF aged 80 years and over were recruited, and 3165 patients were included in the analysis. The flow of participants through each stage of selection based on exclusion criteria is shown in Fig. 1. The mean age was 85.3 ± 4.3 years; 1342 (42.4%) were females (Table 1). Of all participants, 2138 (67.6%) were not prescribed OACs. Patients without OACs were older and more likely to be male than those prescribed OACs. The average HAS-BLED score was significantly higher among patients without OACs than among those prescribed OACs (4.1 vs. 3.9). Patients without OACs had higher comorbidity scores and a higher prevalence of cognitive impairment. The prevalence of antiplatelet use was 48.1% among patients without OACs.

**Table 1:** Demographic and clinical characteristics of the study population

Characteristics	Characteristics of the sample Count (%)/Mean(± SD) *N* = 3165	Prevalence within subgroup Count (%)/Mean(± SD)		*P* value
		**No Anticoagulation** ***N* = 2138 (67.6%)**	**Anticoagulation** ***N* = 1027(32.4%)**	
**Age(years), Mean**	85.3(4.3)	85.7(4.5)	84.3(3.5)	** < 0.001**
**Age(years), N(%)**				** < 0.001**
** 80–84**	1588(50.2)	977(45.7)	611(59.5)	
** 85–89**	1083(34.2)	761(35.6)	322(31.4)	
** 90 + **	494(15.6)	400(18.7)	94(9.2)	
**Sex, N(%)**				** < 0.001**
** Male**	1824(57.6)	1301(60.9)	523(50.9)	
** Female**	1341(42.4)	837(39.1)	504(49.1)	
CHA2DS2-VASC Score*, Mean	4.1(1.3)	4.1(1.3)	4.2(1.3)	**0.006**
CHA2DS2-VASC*, Median	4(3–5)	4(3–5)	4(3–5)	**0.007**
HAS- BLED Score*, Mean	4(1.2)	4.1(1.3)	3.9(1.1)	** < 0.001**
HAS- BLED Score*, Median	4(3–5)	4(3–5)	4(3–5)	** < 0.001**
**HAS- BLED Score, N(%)**				** < 0.001**
** HAS- BLED Score < 3**	308(9.7)	205(9.6)	103(10)	
** 3 < = HAS- BLED Score < = 4**	1771(56)	1148(53.7)	623(60.7)	
** HAS- BLED Score > = 5**	1086(34.3)	785(36.7)	301(29.3)	
**Clinical department, N(%)**				** < 0.001**
** Cardiology**	786(24.8)	383(17.9)	403(39.2)	
** Geriatrics**	1129(35.7)	834(39)	295(28.7)	
** Neurology**	311(9.8)	169(7.9)	142(13.8)	
** Others**	939(29.7)	752(35.2)	187(18.2)	
** Use of antiplatelets, Yes, N(%)**	1392(44)	1028(48.1)	364(35.4)	** < 0.001**
** ASA, Yes, N(%)**	739(23.3)	504(23.6)	235(22.9)	0.7
** Clopidogrel, Yes, N(%)**	1014(32)	767(35.9)	247(24.1)	** < 0.001**
CCI*, Mean	1.9(2)	2.1(2.1)	1.6(1.7)	** < 0.001**
CCI*, Median	1(1–3)	2(1–3)	1(0–2)	** < 0.001**
**CCI**				** < 0.001**
** CCI: 0**	687(21.7)	424(19.8)	263(25.6)	
** CCI: 1–2**	1663(52.5)	1096(51.3)	567(55.2)	
** CCI: 3 + **	815(25.8)	618(28.9)	197(19.2)	
** Hypertension, Yes, N(%)**	2168(68.5)	1460(68.3)	708(68.9)	0.743
** Diabetes, Yes, N(%)**	859(27.1)	570(26.7)	289(28.1)	0.404
** Stroke, Yes, N(%)**	1065(33.6)	706(33)	359(35)	0.299
** CHD, Yes, N(%)**	1393(44)	934(43.7)	459(44.7)	0.62
** Thromboembolic disease, Yes, N(%)**	876(27.7)	576(26.9)	300(29.2)	0.196
** Dementia, Yes, N(%)**	283(8.9)	229(10.7)	54(5.3)	** < 0.001**
**Year of hospitalization, N(%)**				** < 0.001**
** 2016–2017**	722(22.8)	571(26.7)	151(14.7)	
** 2017–2018**	826(26.1)	589(27.5)	237(23.1)	
** 2018–2019**	851(26.9)	548(25.6)	303(29.5)	
** 2019–2020**	766(24.2)	430(20.1)	336(32.7)	

Data are shown using count (percentage) or mean (standard deviation). *P* values were calculated with chi-squared tests and Student's t tests for categorical and continuous variables, respectively

^*^ These variables are also presented as medians (interquartile ranges), and their *p* values were calculated with the Wilcoxon rank sum test

**Fig. 2:**
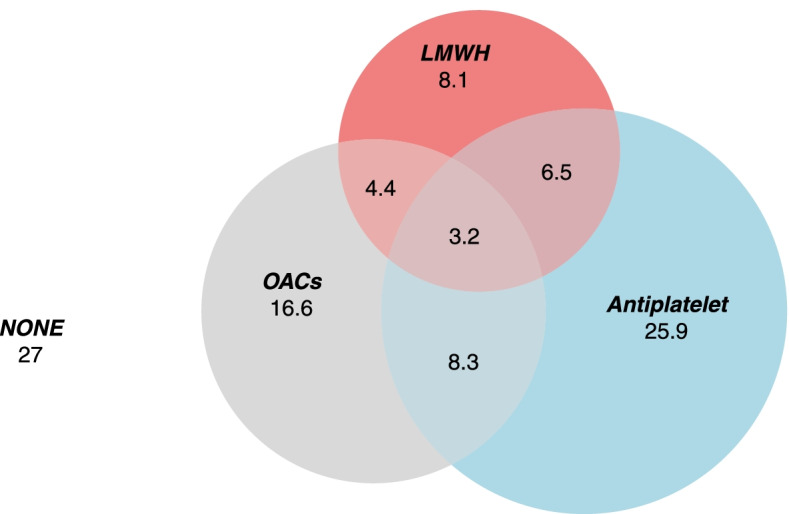
Venn diagram of antithrombotic therapy in patients aged 80 and older with NVAF

Of all patients, 854 (27.0%) did not use any antithrombotic medications (OACs, antiplatelets or LMWH) (Fig. 2). Of the patients without OACs, 25.9% were prescribed antiplatelet agents only, 8.1% were prescribed LMWH only, and 6.5% used LMWH and antiplatelets simultaneously.

### Predictors for anticoagulation therapy use

#### Model 1

In univariate analysis, age, sex, clinical department from which patients were discharged, CHA_2_DS_2-_VASc, use of antiplatelets, use of clopidogrel, CCI, dementia and the year of hospitalization were significantly associated with underprescription of OACs among NVAF patients (Table 2). Patients aged 85–89 and ≥ 90 years had higher odds of not using OACs (OR = 1.48 with 95% CI (1.25–1.74) and OR = 2.66 with 95% CI (2.09–3.42), respectively) than those aged 80–84 years. The odds ratios of not using OACs among patients discharged from geriatric, neurological, and other departments were 2.97 (95% CI: 2.45–3.61), 1.25 (95% CI: 0.96–1.63), and 4.23 (95% CI: 3.43–5.24), respectively, compared to those discharged from the cardiology department. The use of antiplatelets was associated with higher odds of not using OACs (OR = 1.69, 95% CI: 1.45–1.97). Patients with a CCI ≥ 3 were associated with a higher odds of not using OACs (2.02, 95% CI: 1.62–2.53) than those with a CCI = 0.

**Table 2:** Factors associated with underprescription of oral anticoagulants

Characteristics	Model1 [OR(95%CI)] *N* = 3165	Model2 [OR(95%CI)] *N* = 3165	Model3 [OR(95%CI)] *N* = 3165
**Age(years),80–84**	Ref	-	-
**Age(years),85–89**	**1.48(1.25,1.74)**	-	-
**Age(years),90 + **	**2.66(2.09,3.42)**	-	-
**Sex, Female**	**0.67(0.57,0.78)**	-	-
**Clinical department, Cardiology**	Ref	Ref	-
**Clinical department, Geriatrics**	**2.97(2.45,3.61)**	**2.2(1.78,2.71)**	-
**Clinical department, Neurology**	1.25(0.96,1.63)	1.21(0.92,1.57)	-
**Clinical department, Others**	**4.23(3.43,5.24)**	**4.2(3.39,5.21)**	-
**Year, 2016–2017**	Ref	Ref	-
**Year, 2017–2018**	**0.66(0.52,0.83)**	**0.65(0.51,0.83)**	-
**Year, 2018–2019**	**0.48(0.38,0.6)**	**0.47(0.37,0.6)**	-
**Year, 2019–2020**	**0.34(0.27,0.42)**	**0.32(0.25,0.4)**	-
**CHA2DS2-VASC Score**	**0.92(0.87,0.98)**	0.94(0.88,1)	1(0.93,1.07)
**HAS- BLED Score < 3**	Ref	Ref	Ref
**3 < = HAS- BLED Score < = 4**	0.93(0.71,1.19)	0.86(0.66,1.11)	1.11(0.84,1.46)
**HAS- BLED Score > = 5**	1.31(1,1.72)	1.08(0.82,1.43)	**1.89(1.38,2.57)**
**Prescription of antiplatelets, Y**	**1.69(1.45,1.97)**	**1.57(1.35,1.84)**	**2.21(1.85,2.63)**
**Clopidogrel, Y**	**1.77(1.49,2.09)**	**1.59(1.34,1.89)**	**2.1(1.75,2.54)**
**CCI, 0**	Ref	Ref	Ref
**CCI 1–2**	1.2(0.99,1.44)	1.08(0.89,1.3)	1(0.82,1.23)
**CCI 3 + **	**2.02(1.62,2.53)**	**1.68(1.34,2.11)**	**1.39(1.09,1.78)**
**Dementia, Y**	**2.16(1.6,2.96)**	**1.68(1.24,2.33)**	**1.48(1.07,2.07)**
**Thromboembolic disease, Y**	1.12(0.86,1.47)	1.14(0.88,1.5)	0.74(0.56,1)
**Stroke, Y**	0.92(0.78,1.07)	**0.83(0.71,0.98)**	0.89(0.74,1.08)
**Hypertension, Y**	0.97(0.83,1.14)	0.96(0.81,1.13)	0.98(0.82,1.16)
**Diabetes, Y**	0.93(0.79,1.1)	0.91(0.77,1.08)	0.97(0.82,1.17)
**CHD, Y**	0.96(0.83,1.12)	0.87(0.74,1.01)	1.01(0.86,1.19)

Model 1: Univariate logistic regression analysis between no anticoagulation and characteristic variables

Model 2: Multivariate logistic regression analysis between no anticoagulation and characteristic variables adjusted by age and sex

Model 3: Multivariate logistic regression analysis between no anticoagulation and characteristic variables adjusted by age, sex, clinical department and year

#### Model 2

Clinical department, the year of hospitalization, use of antiplatelets, use of clopidogrel, CCI, dementia and prior stroke were associated with underprescription of OACs in multivariable logistic regression analysis adjusted by age and sex (Table 2). 

#### Model 3

In multivariable logistic regression analysis adjusted by age, sex, clinical department and the year of hospitalization, the use of antiplatelets, the use of clopidogrel, CCI and dementia were associated with underprescription of OACs. In particular, the difference in the influence of the HAS-BLED score in model 3 was significant (Table 2).

### Primary versus secondary prevention of stroke in elderly patients with NVAF

Overall, 706 (66.3%) of patients with a history of stroke and 1432 (68.2%) of patients without a history of stroke were not prescribed OACs. A total of 195 (18.3%) of patients with a history of stroke and 659 (31.4%) of patients without a history of stroke were not receiving any antithrombotic therapy. Of the patients with a history of stroke and without OACs, 377 (53.4%) were prescribed antiplatelet agents only, 76 (10.8%) were prescribed LMWH only, and 58 (8.2%) used LMWH and antiplatelets simultaneously. Of the patients without a history of stroke and without OACs, 444 (31.0%) were prescribed antiplatelet agents only, 180 (12.6%) were prescribed LMWH only, and 149 (10.4%) used LMWH and antiplatelets simultaneously.

**Fig. 3:**
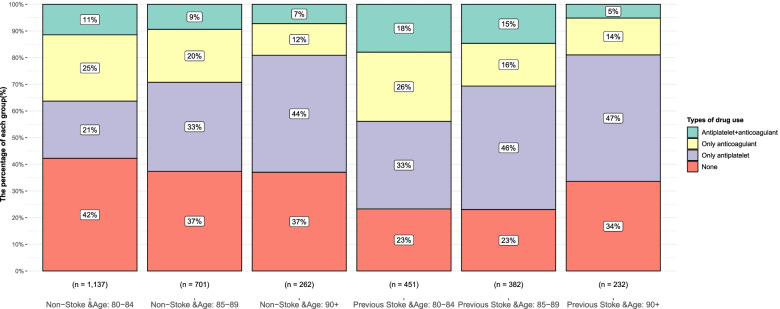
Antithrombotic therapy in nonstroke and previous stroke according to age

Approximately 80.9% of patients ≥ 90 years without a history of stroke were not receiving OACs compared to those aged 80–84 and 85–89 years (63.7% and 70.8%, respectively). Of participants aged 90 years or above, 37.0% were not prescribed any antithrombotic medication (including antiplatelets or OACs) for primary prevention compared to patients aged 80–84 and 85–89 years (42.2% and 37.4%, respectively; Fig. 3). In secondary prevention, patients ≥ 90 years had a higher antiplatelet monotherapy percentage (47.4%) versus 80–84 and 85–89 years (32.8% and 46.3%), and approximately 81.2% of patients 90 years and over were not receiving OACs.

**Fig. 4:**
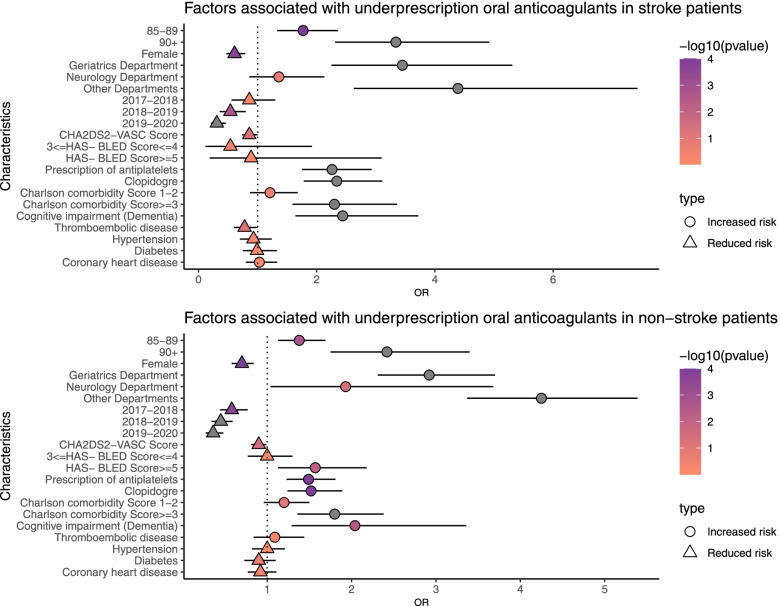
Factors associated with nonuse of oral anticoagulants in nonstroke and previous stroke

As shown in Fig. 4 and Table 3, age, sex, clinical departments from which patients were discharged, years of hospitalization, use of antiplatelets, CHA2DS2-VASC score, CCI, and dementia were associated with underprescription of OACs in primary and secondary prevention. Significant differences in years of hospitalization and HAS-BLED scores were found between the anticoagulation and nonanticoagulation groups in primary prevention.

**Table 3:** Comparison of the prescription of anticoagulants in primary prevention and secondary prevention of stroke

	**Primary prevention**			**Secondary prevention**		
**Characteristics**	**Total *N* = 2100** **Characteristics of the sample (%)**	**Prevalence** **within subgroup (%)**		**Total *N* = 1065** **Characteristics of the sample (%)/Mean(± SD)**	**Prevalence** **within subgroup (%)**	
		**No Anti. *N* = 1432**	**Anti. *N* = 668**		**No Anti. *N* = 706**	**Anti** ***N* = 359**
Age, *N*(%)***###						
** 80–84**	1137(54.1)	724(50.6)	413(61.8)	451(42.3)	253(35.8)	198(55.2)
** 85–89**	701(33.4)	496(34.6)	205(30.7)	382(35.9)	265(37.5)	117(32.6)
** 90 + **	262(12.5)	212(14.8)	50(7.5)	232(21.8)	188(26.6)	44(12.3)
Sex, Female, *N*(%)***###	901(42.9)	574(40.1)	327(49)	440(41.3)	263(37.3)	177(49.3)
Clinical department***###						
** Cardiology, *N*(%)**	679(32.3)	335(23.4)	344(51.5)	107(10)	48(6.8)	59(16.4)
** Geriatrics, *N*(%)**	619(29.5)	458(32)	161(24.1)	510(47.9)	376(53.3)	134(37.3)
** Neurology, *N*(%)**	46(2.2)	30(2.1)	16(2.4)	265(24.9)	139(19.7)	126(35.1)
** Others, *N*(%)**	756(36)	609(42.5)	147(22)	183(17.2)	143(20.3)	40(11.1)
Year, *N*(%)***###						
** 2016–2017**	497(23.7)	397(27.7)	100(15)	225(21.1)	174(24.6)	51(14.2)
** 2017–2018**	567(27)	396(27.7)	171(25.6)	259(24.3)	193(27.3)	66(18.4)
** 2018–2019**	552(26.3)	354(24.7)	198(29.6)	299(28.1)	194(27.5)	105(29.2)
** 2019–2020**	484(23)	285(19.9)	199(29.8)	282(26.5)	145(20.5)	137(38.2)
HAS-BLED Score,*N*(%)**##						
** HAS-BLED Score < 3**	297(14.1)	197(13.8)	100(15)	11(1)	8(1.1)	3(0.8)
** 3 < = HAS- BLED Score < = 4**	1386(66)	920(64.2)	466(69.8)	385(36.2)	228(32.3)	157(43.7)
** HAS- BLED Score > = 5**	417(19.9)	315(22)	102(15.3)	669(62.8)	470(66.6)	199(55.4)
Use of APT, Y, *N*(%)***###	808(38.5)	593(41.4)	215(32.2)	584(54.8)	435(61.6)	149(41.5)
Dementia, Y, *N*(%)**###	115(5.5)	93(6.5)	22(3.3)	168(15.8)	136(19.3)	32(8.9)

Primary prevention, ***p < 0.001, **p < 0.01, *p < 0.05 for comparison of anticoagulation status (anticoagulation, no anticoagulation) within each variable

Secondary prevention, ^###^*p* < 0.001, ^##^*p* < 0.01, ^#^*p* < 0.05 for comparison of anticoagulation status (anticoagulation, no anticoagulation) within each variable

## Discussion

This study aimed to understand the prevalence of underprescription of anticoagulation therapy and to examine the factors associated with underprescription of OACs in NVAF inpatients aged 80 years or older. We found that only 64.9% of older patients with NVAF were prescribed antithrombotic medications, and the prevalence of OAC use was particularly low among those who were older, prescribed antiplatelets, discharged from a noncardiology department, and suffered from comorbidities. OAC use is recommended by clinical guidelines for reducing the risk of stroke among older patients with AF. However, our findings suggest that OACs are still severely underprescribed.

We found that less than one-third of NVAF patients aged ≥ 80 years and less than one-fifth of those aged ≥ 90 years were prescribed OACs. Although the prevalence gradually increased from 2017 to 2020 (20.9% in 2017, 28.7% in 2018, 35.6% in 2019 and 43.9% in 2020), this prevalence was lower than that in several previous studies showing that 37.5–58.5% of NVAF elderly patients receive OACs [4, 7, 18, 19]. The FIELD GARFIELD registry reported that nearly 60% of NVAF patients with a high risk of stroke received OACs in China in 2016. In our study, we focused on a sample of older adults with an average age of approximately 85 years.

We found that the prevalence of underprescription of OACs was particularly high among patients who were taking antiplatelets. These results were in line with previous studies [5, 6, 18, 20–23]. Our results showed that 44.0% of NVAF patients aged ≥ 80 years were prescribing antiplatelets, and 25.9% of patients received antiplatelet monotherapy. Slightly over one-third of patients without atherosclerotic disease were prescribed antiplatelets, and we strongly suspect that these prescriptions were made to prevent the thromboembolic complications of NVAF. To date, the largest cohort study involving participants aged ≥ 90 years was conducted in Taiwan, which included 15,756 participants [24]. As a result, OACs were superior in reducing the risk of ischemic stroke and positive net clinical benefit with no significant difference in safety compared with aspirin in patients with NVAF ≥ 90 years.

Although NVAF patients who experienced ischemic stroke are likely to have a recurrence, we did not find the utilization of anticoagulants in patients with a stroke history to be higher than those without. Underprescription of OACs in secondary prevention of stroke in octogenarian patients was mainly related to antiplatelet prescription, as was pronounced in patients ≥ 85 years. More than 46.7% of patients aged 85 years and older were only prescribed antiplatelets in secondary prevention versus 36.2% in primary prevention, which is in line with community-based contemporary registry data that one-third of NVAF patients with high stroke risk did not receive OACs but instead were prescribed antiplatelet monotherapy even untreated [25]. Indeed, antiplatelets do not significantly reduce ischemic stroke risk and have no impact on mortality [26].

In this study, the majority of patients aged ≥ 80 years with NVAF were not discharged from the Department of Cardiology (24.8%) or Neurology (9.8%). Older patients with NVAF, accompanied by multiple comorbidities, were likely to be admitted to medical specialties such as the Department of Geriatrics Medicine, Respiratory, Nephrology, Oncology, and surgical departments, in which most physicians have not always been trained in standard anticoagulation therapy for NVAF. Previous studies and systematic reviews indicated no interaction for age with regard to both the efficacy and safety of OACs, even in patients aged ≥ 75 years [27–30].

Despite recommendations of guidelines or consent, the proportion of NOACs prescribed in our older NVAF population taking OACs was only 60.9% (39.1% for warfarin, 28.1% for dabigatran, 28.7% for rivaroxaban, and 2.7% changed warfarin into NOACs, 0.6% changed NOACs into warfarin, and 0.8 interchanged between NOACs) due to higher cost. The proportion of NOACs prescribed in our older NVAF population taking OACs was increasing year by year (13.2% in 2017, 50.6% in 2018, 73.3% in 2019, 78.6% in 2020), which should be the main reason why the use of anticoagulants was increasing year by year.

We acknowledge several limitations. First, except for geriatricians, most physicians in other departments did not implement comprehensive geriatric assessment (CGA) for older patients in their clinical practice in China, and we could not assess the health status of the elderly more fully. Second, anticoagulation therapy in NVAF patients may also be associated with the risks for stroke, physicians’ perception, patients’ preferences, and patients’ attitudes toward anticoagulation for NVAF. These data were not available in some cases due to a lack of medical records. Finally, our study was conducted in a teaching hospital, and therefore, the results may not represent other hospitals in different regions.

## Conclusions

This study demonstrated that the prevalence of underprescription of OACs among older NVAF inpatients is high and associated with age, prescription of antiplatelets, department from which patients are discharged, comorbidities, hypertension, history of stroke, and cognitive status. Further research is needed to confirm and modify the other factors related to nonadherence to guideline-directed anticoagulation therapy in very older NVAF patients.

## Data Availability

The data that support the findings of this research are available on request from the corresponding author. The data are not publicly available due to privacy or ethical restrictions.

## Abbreviations

NVAF: Nonvalvular atrial fibrillation
AF: Atrial fibrillation
DOACs/NOACs: Direct and nonvitamin
K: Antagonist oral anticoagulants
CI: Confidence interval
CHD: Coronary heart disease
INR: International normalized ratio
CCI: Charlson comorbidity index
TIA: Transient ischemic attack
OACs: Oral anticoagulants
LMWH: Lower molecular weight heparins
